# Low climate-patterned temperature and cardiovascular disease: Worldwide trends and implications for public health policy

**DOI:** 10.1016/j.ijcrp.2025.200437

**Published:** 2025-05-22

**Authors:** Wenpeng You, Jacob Sevastidis, Frank Donnelly

**Affiliations:** a School of Biomedicine, The University of Adelaide, Adelaide, Australia; b Adelaide Nursing School, The University of Adelaide, Adelaide, Australia; c Heart and Lung, Royal Adelaide Hospital, Adelaide, Australia; d School of Nursing and Midwifery, Western Sydney University, Sydney, Australia

**Keywords:** Cardiovascular disease incidence, Cold temperature, Climate-patterned temperature, Long-term pattern of mean value of temperature, Population health

## Abstract

**Background:**

Short-term cold spells and heat events are commonly considered risk factors for cardiovascular disease (CVD). This study quantitatively examined the effects of country-specific "climate-patterned temperature" (T_MP_), measured as long-term mean temperature, on global CVD incidence.

**Methods:**

Recently published country-specific data on CVD incidence and T_MP_ were analysed for statistical correlations at the population level using Microsoft Excel and SPSS. Confounding effects of humidity, aging, GDP PPP, obesity prevalence, and urbanization were controlled. Fisher r-to-z transformation compared correlation coefficients.

**Results:**

Pearson's r and nonparametric analyses revealed a significant inverse correlation between T_MP_ and CVD incidence worldwide (r = −0.646 and −0.574, respectively, p < 0.001). This relationship remained significant after controlling for confounders in a partial correlation model (r = −0.584, p < 0.001). Multiple linear regression showed T_MP_ as a significant and independent predictor of CVD incidence (Beta = −0.384, p < 0.001). Stepwise regression identified aging as the most influential factor (R^2^ = 0.591), with T_MP_ and GDP PPP following, increasing R^2^ to 0.731 and 0.747, respectively. Humidity, obesity prevalence, and urbanization were not significant predictors. T_MP_ had a stronger predictive effect on CVD incidence in high-income countries compared to low- and middle-income countries (z = 1.96 and 2.28 in Pearson's r and nonparametric models, respectively, p < 0.05).

**Conclusions:**

Long-term lower mean temperature (T_MP_) is a significant and independent risk factor for CVD worldwide, particularly in developed countries. T_MP_ should be considered in epidemiological studies of CVD.

## Highlights

• Lower average ambient temperatures link to higher rates of heart disease worldwide.
• Cold climate impacts heart disease risk, even with lifestyle factors controlled.
• Cold climates predict heart disease more strongly in wealthier countries.
• Considering temperature can improve heart disease prevention in colder regions.

## 1 Introduction

Cardiovascular diseases (CVDs) are chronic conditions with lifelong implications, affecting the heart and blood vessels [1]. Globally, CVD caused 17.9 million deaths in 2019, marking a 24 % increase since 1990 [2]. CVD represents over 60 % of the lifetime risk for global morbidity and mortality [1]. Over the past three decades, CVD has rapidly emerged as the leading cause of mortality worldwide [2]. While numerous studies underscored the escalating burden of CVD on healthcare systems, some indicated an overall declining trend in global CVD incidence and mortality [3,4]. This emphasizes the urgent need for enhanced understanding and prevention of CVD, making it a crucial public health priority in both developed and developing countries [5].

CVDs have multiple aetiologies driving the worldwide increase of CVD incidence [6]. Therefore, approaches to diagnosing and preventing CVD which have traditionally focused on the assessment and treatment of lifelong key risk factors should also consider both genetic and environmental factors [6,7]. Family linkage studies have shown that CVD development involves strong genetic underpinnings [8]. It has been well-established that at a population level, ageing [9], urban living [10,11], obesity and overweight [[12], [13], [14]] and economic affluence [15,16] have been recognized as the major environmental risk factors for CVD.

It is well-known that variations in ambient temperature change have been associated with increased CVD mortality worldwide because of the human biological response to extremes of thermal environments [[17], [18], [19]]. The rationale for the associations is that ambient temperature change can cause blood vessels to contract or dilate which may lead to fluctuations in blood pressure [18,20], cardiac hypertrophy [20], increased blood viscosity [21], and subsequently CVDs [22,23]. A number of studies have examined the role of extreme temperatures in contributing to CVDs, for example coronary heart disease [24], ischemic heart disease [25], myocardial infarction [24], cerebrovascular disease [26], heart failure [25], stroke [24,25], and total CVDs [17,18].

Weather refers to short-term atmospheric conditions. Severe weather changes, such as heat waves and cold spells have adverse effects on human health, which have been extensively associated with CVD mortality at population level in a large body of research [[27], [28], [29], [30], [31], [32]]. For example, in the past decades, severe heat waves have shown significant impacts on excess mortality, such as incidents in Chicago (1995), France and broadly Europe (2003). In contrast, cold spells leading to large numbers of deaths have occurred in Czech Republic (1987) and Moscow (2006).

Meteorologically, climate-patterned temperature, indicated by the averaged temperature over a specific region for 30 years or more (T_*MP*_), reflects a temperature pattern aligned with the long-term climate features of the area. Compared to short-term weather changes, for example extreme heat and cold spells, T_*MP*_ has not been systematically associated with CVD development. Given the complexity of CVD pathology, this study has identified a number of opportunities to reflect on biases in the existing research literature:

1. Curiously, a large number of studies, rather than considering the total CVD impact, have focused on individual CVDs, such as coronary heart disease [24], myocardial infarction [24], cerebrovascular disease [26], heart failure [25], stroke [24,25] and their association with short-term temperature changes. These studies have provided more reliable and consistent data, recognizing potential variations in the temperature's impact on specific cardiovascular diseases (CVDs) and their temperature-dependent susceptibility. Nonetheless, each CVD demonstrated interconnections with other types of CVDs, representing comorbidities [33], which may lead to excessive incidence. As the cumulative incidence of total CVDs has consistently been documented at the population level [2] there is an opportunity to examine the impact of temperature on the comprehensive incidence of CVDs.
2. Previous studies have tended to consider the impact of short periods of time such as days to weeks of temperature extremes without consideration of the impact of much longer durations of exposure [25], e.g. across decades. This may not have allowed the authors to explore if the long-term temperature (climatically patterned) had a predicting role in CVD morality or incidence.
3. The associations between extreme temperature and CVDs have been examined within relatively small regions, with no study examining the relationship across-country environments [25,32]. Due to regional CVD variation, analyses of only small regions may restrict insight into the association between extreme temperature change and total CVD mortality [34].
4. Previous studies have also not considered a range of major confounding factors when exploring the relationship between extreme temperature and CVD risk [4,25,35].

Moreover, the Earth's temperature has increased by an average of 0.06 °C per decade since 1850, with a more rapid rise observed in recent decades [36]. Concurrently, with the recent accelerated temperature increase, some studies have suggested an overall decline in both CVD incidence and CVD mortality worldwide [3,4]. These findings challenged the notion that global warming is a culprit in increasing modern disease burden, including CVD development.

Given the above potential study biases and challenges in explaining the temperature- CVD relationship in previous research, in this cross-sectional study it was hypothesized that countries with long-term patterns of lower mean temperature (T_*MP*_) had greater cardiovascular disease incidence rates. This hypothesis was examined through analysing the statistical correlation between T_*MP*_ and total CVD incidence at population level. The well-established potential confounding factors for the T_*MP*_ – CVD relationship, such as humidity, ageing, economic affluence, obesity and urban living were incorporated into the data analyses for exploring the independent and significant role of T_*MP*_ in predicting CVD incidence.

## 2 Material and method

### 2.1 Data sources and selection

To analyze the role of T_*MP*_ in determining CVD incidence worldwide, the statistical role of T_*MP*_ in determining CVD incidence was analysed at three levels, i.e. confounded T_*MP*_-CVD correlation, independent role of T_*MP*_ in determining CVD and revealing T_*MP*_ as the second most influential factor for CVD incidence. The country level data published by several international organizations were captured for achieving these study goals in this ecological study. Referencing previous studies [37,38], the role of T_*MP*_ in determining CVD incidence was examined with the following variables:

The dependent variable is the country specific CVD incidence rate. Published by the Institute for Health Metrics and Evaluation of the University of Washington [39,40], the estimate of CVD incidence rate is expressed as the number of newly diagnosed CVD cases per 100,000 in 2017. We could not include the ICD codes in our dataset for subsequent analysis because they are not provided by IHME when they published their CVD data.

The independent variable is the country specific long-term pattern of mean value of temperature (Celsius, average yearly temperature for 30 years, 1988–2017, T_*MP*_ hereafter) [41,42]. For each country/population, T_*MP*_ was downloaded from the World Bank Group Climate Change Knowledge Portal [41]. The average temperature for 30 years was then calculated. The rationale for capturing this independent variable is that, typically, meteorological science adopts the average of a temperature over a period of 30 years or more for indexing a genuine temperature pattern for a region [42].

CVDs encompass multiple aetiologies, involving risk factors such as smoking, obesity, blood pressure, serum lipids, fasting glucose, dietary pattern, physical activity, diabetes, age, and kidney dysfunction [43,44]. These factors may compete with low T_*MP*_ in contributing to CVD incidence. Due to data availability constraints at the population level, not all relevant risk factors were accessible for our ecological study. A literature review identified some key variables recognized as significant CVD risk factors throughout the lifespan [45]. These were included in our data analyses as potential confounders.

1) Humidity indexed with average annual precipitations for the same period (1988–2017) which was also used for calculating the climate-patterned temperature (T_*MP*_) [41].

The stress temperature threshold for CVD pathogenesis remains uncertain and likely varies across different regions, however can be considered alongside humidity [46]. For instance, stress temperature thresholds may become higher when humidity is lower, and vice versa [46]. In other words, the combination of effects of the specific sets of temperature and humidity may have different effects on CVD pathogeneses [46].

2) Ageing expressed with life expectancy at 65 years old in 2014 (ageing) [[47], [65]].

Published by the United Nations, an increasing average life expectancy may indicate a higher level of healthcare service, nutritional improvements and a decrease in infectious disease mortality. Individuals aged 65 or older are more susceptible to CVD diseases due to a reduced capacity for thermoregulation and low physiological tolerance to ambient temperature changes [9,48,49]. Additionally, elderly people are more likely to have developed pre-existing conditions of CVDs such as hypertension, diabetes mellitus, hyperlipidaemia and coronary artery disease and obesity [50]. Cold stressors may be worse for the elderly because of co-morbidities and lower capacity for adaptive responses to unfavourable external stimulus [17,51].

3) GDP PPP expressed in per capita purchasing power parity (US dollars) in 2014 published by the World Bank [15,16].
GDP PPP as a major indicator of socioeconomic status determines not only people's access to primary healthcare services that include CVD screening [16,52], but also their affordability to maintain comfortable temperature ranges through access to heating and cooling technologies [53].
4) Obesity prevalence rate (adult) in 2014 published by the WHO Global Health Observatory (GHO) [54].

Obesity prevalence indicates the percentage of population aged 18+ with BMI ≥30 kg/m^2^ [54]. Posing multifactorial health challenges, obesity contributes directly to CVD incidence, and indirectly contributes to CVD comorbidities, such as hypertension, type 2 diabetes, dyslipidaemia, and atherosclerosis in both adults and children [[12], [13], [14]].

5) Urbanization is expressed with the percentage of a population living within urban areas in 2014, as published by the World Bank [15].

Urbanization often entails a high level of education and healthcare services, yet may also encourage poor lifestyle choices, such as lack of physical exercise and social engagement, more intake of less nutritional food, high levels of gluten and processed meats, salt, fat, sugar and alcohol and lower vegetable consumption. Urban lifestyle has been recognized as a complex risk factor for chronic diseases, including CVDs [10,11,55].

The World Bank Group maintains a powerful database including collections of time series data on a variety of topics, such as socioeconomics, health, climate and demography [56] with 217 geographic locations/territories contributing their data. For the purposes of this paper, regardless of the political independent sovereign, geographic location/territory is interchangeably called country or population [57].

Firstly, a list of 217 geographic country locations was downloaded and all the above 7 variables were matched against each country. We considered each country/population an individual study subject for each data analysis model. However, not all the geographic locations/territories provided information for all relevant variables in the World Bank database. Therefore, the numbers of countries/population included for our analyses may differ in the different data analysis models used.

The diagnosis of CVD as a health condition may be a consequence of delay in the presentation of risk factors. In other words, CVD presentation may only occur after a cumulative exposure to the risk factors, including the independent variable (T_*MP*_) and potential confounding variables (humidity, ageing, GDP PPP, obesity and urbanization). For instance, if a population is exposed to higher GDP PPP, CVD incidence in this population may not increase immediately, but over several years. Therefore, country specific data on ageing, GDP PPP, obesity and urbanization were backdated 3 years from 2017 to account for an accumulation of confounding effects on CVD incidence. To represent the potential influences of climate, the long-term climate pattern of temperature and concurrent humidity were then incorporated for data analyses.

### 2.2 Data analysis

To examine the statistical T_*MP*_-CVD relationship for illustrating the role of T_*MP*_ in predicting CVD incidence, the analysis proceeded in 5 steps after referring to the previous studies authored by You et al. [[58], [59], [60]].

1) Bing© was used to integrate countries and their respective T_*MP*_s into the world geographic map in Microsoft Excel® 2016, and each country was colour saturated depending on their T_*MP*_ values. Intuitively, the T_*MP*_s of different countries can be visualised depending on the darkness of colour and their latitudes.

With the raw data, scatter plots were also prepared for exploring and visualizing the correlation between T_*MP*_ and CVD incidence in Excel (Microsoft® 2016). Scatter plots allowed an observation of correlation between T_*MP*_ and CVD incidence and an examination of the variable distributions and data quality, such as identifying any major outliers. In this study, Greenland was identified as an outlier (T_*MP*_ = - 18.36 °C, CVD incidence = 1590.84 per 100,000) and was removed from the following data analyses.

Before running correlation analyses, all above 7 variables were log-transformed to reduce possible curvilinearity of regressions and data non-homoscedasticity due to their abnormal distributions. The T_*MP*_s in the Russian Federation (-3.92 °C) and Canada (-3.59 °C) were below zero. The negatives cannot be log-transformed for exploring the best fit trendline because logarithmic, polynomial and power trendlines could not be drawn and visualised. Therefore, 5 °C was added to T_*MP*_ for each country for identifying the best trendline between T_*MP*_ and CVD incidence.

2. Bivariate correlations (Pearson's and nonparametric) were used to quantify the strength and direction of the correlations between all variables. This common approach cross-checks aligning correlations with a-priori data and allowed us to ensure data quality. This allowed us for example, to check if the potential confounding variables were properly chosen.
3. Partial correlation of Pearson's moment-product correlation was performed to examine the correlation between T_*MP*_ and CVD incidence while the competing variables (humidity, ageing, GDP PPP and urbanization) were kept statistically constant.

T_*MP*_ was first incorporated as an independent variable for predicting CVD incidence while humidity, ageing, GDP PPP, obesity and urbanization were kept constant. Then, T_*MP*_ was kept statistically constant to explore the independent predicting effects of humidity, ageing, GDP PPP, obesity and urbanization on CVD incidence respectively. This examined how much T_*MP*_ can explain each independent variable influencing CVD incidence.

4 Standard multiple linear regression (enter model) was conducted to analyze the respective predictive effects of humidity, ageing, GDP PPP, obesity and urbanization on CVD incidence. Subsequently, stepwise linear regression was performed to select the predictor(s) having the best influencing effects on CVD incidence.

To see if and how much T_*MP*_ affected the predicting effects of humidity, ageing, GDP PPP, obesity and urbanization on CVD incidence in both enter and stepwise models, T_*MP*_ was added and omitted as one of predicting variables for both multiple linear regression analyses.

5. The 217 countries were also grouped by different classification criteria; exploring the regional relationships between T_*MP*_ and CVD with Pearson's r and nonparametric tests:
  a) The World Bank income classifications: high income, upper middle income, low-middle income and low income; In response to the WHO's estimate that 75 % of CVD deaths occur in low- and middle-income countries (LMIC) [2] these countries were combined for creating another country grouping. Pearson's r and nonparametric tests were conducted for exploring the correlation between T_*MP*_ and CVD incidence. Fisher's r-to-z transformation was applied for comparing the bivariate correlations of T_*MP*_ to CVD in LMIC and high-income countries.
  b) The developed and developing countries defined with the United Nations' common practice [61]. As a further response to the above WHO statement, Fisher's r-to-z transformation was applied to compare the Pearson's r and nonparametric correlations between T_*MP*_ and CVD incidence in developed countries and in the developing countries.
  c) Countries with a strong contrast in geographic distributions of income levels and/or cultural backgrounds.

We analysed these correlations in 8 country groupings: Asia Cooperation Dialogue (ACD) [62]; the Asia-Pacific Economic Cooperation (APEC) [63]; the Arab World [64], countries with English as the official language (informed through government websites), Latin America [65], Latin America and the Caribbean (LAC) [65], the Organisation for Economic Co-operation and Development (OECD) [66] and Southern African Development Community (SADC) [67].

Bivariate correlations (Pearson's r and nonparametric rho), Pearson's moment-product partial correlation and multiple linear regressions were performed with log-transformed data in SPSS v. 28 (Chicago Il USA) [68]. The significance was reported when p was below 0.05, but the stronger significance levels, such as p < 0.01 and p < 0.001 were also indicated in this study. Regression analysis criteria were set at probability of F to enter ≤0.05 and probability of F to remove ≥0.10.

## 3 Results

**Fig. 1:**
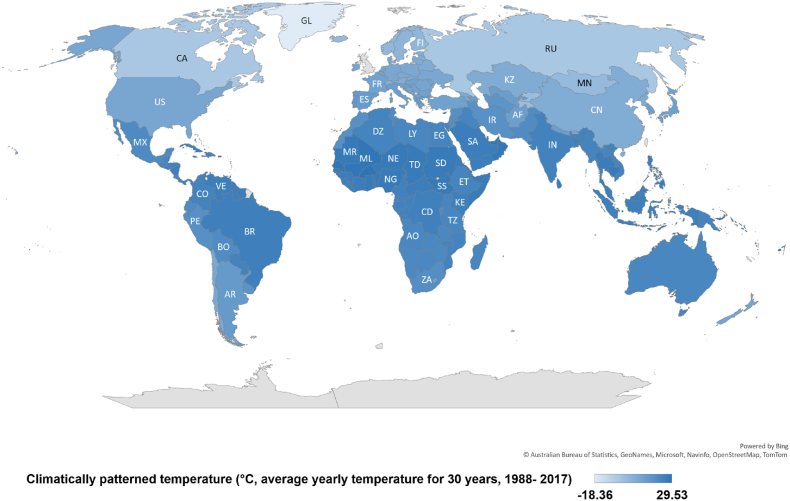
The world map of patterned temperature (TMP) for all countries.

Fig. 1 showed that most of the countries with low T_*MP*_ are located in Europe and North America with high latitudes (north). Worldwide, the lowest T_*MP*_ was in Greenland (-18.36) which was 47.89 °C lower than highest T_*MP*_ in Burkina Faso (29.53). Worldwide, the mean of CVD incidence rate was 19.52 (Fig. 1). Fig. 1 also showed that most LMICs are in warmer regions, while high-income countries are in colder areas.

Data source & definition: Climatically temperature pattern (T_*MP*_), °C, average yearly temperature for 30 years, 1988–2017, the World Bank Group CCKP.Both variables were not log-transformed for correlation analysis.

Fig. 2.1 and 2.2 revealed that, worldwide, T_*MP*_ displayed a strong, negative, and significant correlation to CVD incidence rate. Both figures demonstrated that the T_*MP*_-CVD incidence relationship did not produce J-, V- or U-shaped trendlines. However, there appeared to be an outlier in the data which is Greenland (T_*MP*_ = - 18.36 °C, CVD incidence = 1590.84 per 100,000, Fig. 2.1). After removing the outlier, the best fit T_*MP*_ -CVD trendline was polynomial regression line (R^2^ = 0.4694 (r = − 0.6851), p < 0.001, n = 192, Fig. 2.2).

**Fig. 2:**
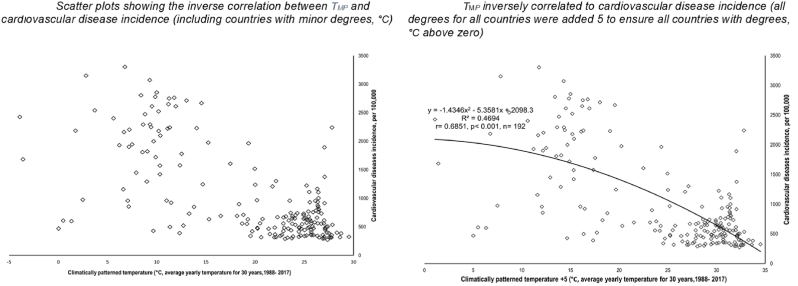
The relationship between temperature pattern (T_*MP*_, °C) and cardiovascular incidence rate.

Data source & definition: Climatically temperature pattern (T_*MP*_), °C, average yearly temperature for 30 years, 1988–2017, the World Bank Group CCKP; Cardiovascular disease (CVD) incidence rate (per 100,000) 2017, the Institute for Health Metrics and Evaluation. Both variables were not log-transformed for correlation analysis.

Table 1 showed that, worldwide, T_*MP*_ was in a strong, negative and significant correlation to the variable of CVD incidence in both Pearson's r and nonparametric analyses (r = − 0.646, p < 0.001 and r = − 0.574, p < 0.001 respectively). Additionally, in both data analysis models, CVD incidence was significant and produced moderate to strong, positive correlations to ageing, GDP PPP, obesity and urbanization respectively (r range: 0.428–0.820, p < 0.001). However, humidity nearly showed nil correlation to CVD incidence. This suggested that humidity may not have a major role in determining the incidence of CVD.

**Table 1:** Pearson's r and nonparametric correlation matrix between all variables.

	CVD incidence	Humidity	T_*MP*_	Ageing e [65]	GDP PPP	Obesity %	Urbanization
CVD incidence	1	−0.014	−0.646∗∗∗	0.764∗∗∗	0.734∗∗∗	0.428∗∗∗	0.526∗∗∗
Humidity	−0.133	1	0.027	0.008	−0.164∗	−0.190∗∗∗	−0.155∗
T_*MP*_	−0.574∗∗∗	0.180∗∗∗	1	−0.391∗∗∗	−0.392∗∗∗	−0.186∗	−0.217∗∗∗
Ageing e [65]	0.782∗∗	−0.061	−0.399∗∗	1	0.811∗∗∗	0.362∗∗∗	0.556∗∗∗
GDP PPP	0.775∗∗∗	−0.202∗∗∗	−0.373∗∗∗	0.820∗∗∗	1	0.502∗∗∗	0.720∗∗∗
Obesity %	0.473∗∗∗	−0.212∗∗	−0.146∗∗	0.388∗∗∗	0.483∗∗∗	1	0.546∗∗∗
Urbanization	0.555∗∗∗	−0.154∗	−0.209∗∗∗	0.604∗∗∗	0.757∗∗∗	0.584∗∗∗	1

Pearson r (above diagonal) and nonparametric (below diagonal) correlations were reported.

Significance levels: ∗P < 0.05, ∗∗∗P < 0.001. Number of country range (sample size): 176–209.

Table 2 suggested that T_*MP*_ is a significant risk factor for CVD incidence regardless of co-variates (humidity, ageing, GDP PPP, obesity and urbanization) influencing CVD incidence (r = 0.584, p < 0.001). However, when T_*MP*_ was statistically kept constant, humidity, ageing, obesity and urbanization showed significant correlations to CVD incidence (r = 0.728, 0.685, 0.410 and 0.518 respectively, p < 0.001).

**Table 2:** Partial correlations between cardiovascular disease incidence and independent variable when temperature pattern was included as the independent and confounder respectively.

Variables	T_*MP*_ partially correlated to CVDs			T_*MP*_ kept constant statistically, other variables partially correlated to CVDs		
	r	p	df	r	p	df
T_*MP*_	−0.584	<0.001	169	–	–	–
Humidity	–	–	–	0.728	<0.001	189
Ageing e [65]	–	–	–	0.685	<0.001	177
GDP PPP	–	–	–	0.004	0.952	189
Obesity %	–	–	–	0.410	<0.001	177
Urbanization	–	–	–	0.518	<0.001	189

All the data were log-transformed for correlation analysis.

- Included as the confounding factor.

Table 3 showed that when T_*MP*_ was not added as a predicting variable in the multiple linear regression (enter), ageing and GDP PPP were the only two variables significantly influencing CVD incidence (Beta = 0.486 and 0.346 respectively). When T_*MP*_ was added as a predicting variable, T_*MP*_, ageing and GDP PPP were the only 3 variables having a significant influence on CVD incidence (Beta = - 0.384, 0.410 and 0.235 respectively). Regardless of T_*MP*_ being a predicting variable or not, humidity, obesity and urbanization showed very weak or almost no correlation to CVD incidence.

**Table 3:** Multiple linear regression analyses sorting significant predictors of cardiovascular disease incidence.

3-1: ENTER		TMP not added		TMP added	
Variables Entered		Beta	Sig.	Beta	Sig.
T_*MP*_		Not applicable	Not applicable	−0.387	<0.001
Ageing e [65]		0.486	<0.001	0.410	<0.001
GDP PPP		0.346	<0.001	0.235	<0.010
Humidity		0.042	0.400	0.033	0.413
Obesity %		0.080	0.165	0.077	0.106
Urbanization		−0.050	0.488	−0.038	0.522

3-2: STEPWISE	T_*MP*_ not added			T_*MP*_ added	
Rank	Variables entered	Adjusted R square	Rank	Variables entered	Adjusted R square

1	Ageing e [65]	0.591	1	Ageing e [65]	0.591
2	GDP PPP	0.626	2	T_*MP*_	0.731
	T_*MP*_	Not added	3	GDP PPP	0.747
	Humidity	Non-significant		Humidity	Non-significant
	Obesity %	Non-significant		Obesity %	Non-significant
	Urbanization	Non-significant		Urbanization	Non-significant

Stepwise multiple linear regression modelling is reported. Contribution of variables is listed in order of how much they contribute to cardiovascular disease incidence.

All the data were log-transformed for correlation analysis.

In the subsequent multiple linear regression (stepwise), when T_*MP*_ was not added as a predicting variable, ageing and GDP PPP were placed first and second most significant predicting variables for CVD incidence respectively (Table 2, Table 3). When T_*MP*_ was added as a predicting variable, it was placed second to have the greatest influence on the CVD incidence (significantly increasing R^2^ from 0.591 to 0.731). Ageing and GDP PPP were placed first and third most influential predictors for CVD incidence (Table 2, Table 3).

In both enter and stepwise models, humidity, obesity and urbanization were weak predictors of CVD incidence.

Table 4 indicated that, in general T_*MP*_ inversely correlated to CVD incidence in different country groupings, even when the strength of the correlation and significance levels varied. One of the highlights of the findings was that T_*MP*_ retained a significantly stronger inverse correlation to CVD incidence in high-income countries compared to low- and middle-income countries (LMIC) (z = 1.96 and 2.28, p < 0.050 in Pearson's r and nonparametric respectively).

**Table 4:** Temperature pattern (T_*MP*_) determined cardiovascular disease incidence in different country groupings.

Country groupings	Pearson r	p	Nonparametric	p
Worldwide (n = 192)	−0.646∗∗	<0.001	−0.574∗∗	<0.001
World Bank income classifications				
High Income, n = 62	−0.724∗∗	<0.001	−0.681∗∗	<0.001
Low Income, n = 28	−0.395∗∗	<0.050	−0.361∗	0.059
Low Middle Income, n = 48	−0.419∗∗	0.050	−0.343∗	<0.050
Upper Middle Income, n = 54	−0.652∗∗	<0.001	−0.401∗∗	<0.010
Low- and middle-income countries (LMIC), n = 130	−0.542∗∗	<0.001	−0.440∗∗	<0.001
Fisher r-to-z transformation	High vs LMIC: z = 1.96, p < 0.050		High vs LMIC: z = 2.28, p < 0.050	
United Nations common practice				
Developed, n = 48	−0.385∗∗	0.010	−0.209	0.154
Developing, n = 144	−0.296∗∗	<0.001	−0.246∗∗	<0.010
Countries grouped with various factors				
English as Official Language, n = 51	−0.561∗∗	<0.001	−0.131	0.358
Asia-Pacific Economic Cooperation, n = 19	−0.658∗∗	<0.010	−0.535∗	<0.050
Organisation for Economic Co-operation and Development, n = 37	−0.519∗∗	<0.001	−0.328∗	<0.050
Arab World, n = 21	−0.256	0.262	−0.512∗	<0.050
Asia Cooperation Dialogue, n = 33	−0.146	0.416	−0.029	0.871
Southern African Development Community, n = 16	0.021	<0.050	−0.041	0.880
Latin America, n = 31	−0.144	0.440	−0.088	0.638
Latin America and Caribbean, n = 34	−0.295	0.090	0.172	0.331

All the data were log-transformed for correlation analysis.

## 4 Discussion

There has been much interest in examining the role of low temperature and increasing CVD incidence. Current literature suggests that short term cold temperatures (cold spells) have detrimental effects on CVDs in a number of studies [69,70]. With multiple data analysis models, our study uniquely suggested that low temperature had negative impact on CVD progenesis at three levels:

1. Consistent with previous studies into the low temperature-CVD relationship, the inverse correlation between T_*MP*_ and CVD incidence in this study suggested that countries with lower T_*MP*_ may have higher CVD incidence rates.
2. Low T_*MP*_ independently predicted CVD incidence once the potential confounding effects of humidity, ageing, GDP PPP, obesity and urbanization on the low temperature-CVD relationship were removed from the model.
3. Second to GDP PPP, T_*MP*_ showed its significantly detrimental effects on CVD progenesis. Statistically, humidity, obesity and urbanization had insignificant or negligible influence on CVD pathogenesis.

In metrological science, "weather" refers to the short-term atmospheric conditions in a specific location, typically observed over a period of less than 30 years. Rapid low temperature changes (i.e. cold spells) have been extensively and almost unanimously postulated as a CVD risk factors due to increases of blood pressure [20,71], blood viscosity [21], platelet [21,71] and red cells counts [72]. It has also been associated with individual CVD types, such as cardiac hypertrophy [20]. However, a large majority of these studies were focused on revealing the risk of short-term low temperature conditions on different individual CVD types in specific locations. For example, the impact of winter temperatures on acute myocardial infarction and recurrent myocardial infarction was studied in Stockholm County, Sweden, from 1990 to 2002 [73]. Similarly, elevated cardiovascular disease risk factors, such as raised systolic blood pressure, were examined across 10 diverse regions in China between 2004 and 2008 [74].

In contrast, the independent variable in our study is the country-specific mean temperature value over 30 years (T_*MP*_). It was informed by the prevailing climate conditions of geographic location of each country, also called “climate-patterned temperature”. Our literature review showed that there has been no substantial research into the detrimental effects of long-term, low climate-patterned temperature on CVDs. As Earth's temperature has been on the rise over the past decades researchers have naturally focused on investigating the correlation between global warming and human health challenges, such as CVDs [75]. Arguably, CVD epidemiology studies have observed a global decrease in both CVD incidence and mortality [3,4]. This suggests a potential protective role of overall global warming in mitigating CVD pathogenesis. This suggestion is compatible with our study findings that countries with higher climate-patterned temperature may have lower CVD incidence rates.

Our analysis incorporated CVD incidence rate as the dependent variable, deviating from previous studies that predominantly used CVD mortality. CVD mortality, representing death due to CVDs, is an indicator of the adverse risk effects of cold temperature encompassing phases before diagnosis and subsequent clinical and non-clinical treatments. Mortality may not accurately predict the negative impact of cold temperature on CVD development, as the temperature-CVD relationship might be confounded by complex bio-psycho-social interventions which were associated with other important factors like economic status, urban advantages, and aging. However, these factors were often omitted in prior research. The inclusion of CVD incidence rate in our study offers a superior risk indicator, capturing new cases over a specified period for the population and less impacted by the complexities of pre-treatment or post-treatment effects.

Concerns have been raised about previous studies suggesting that low (cold) temperature might be seen as a precipitating factor rather than a long-term predisposing factor for CVDs [76]. Since CVDs often exhibit delayed onset, the impact of low temperature and other risk factors may take decades to manifest as symptomatic CVD. Previous studies often correlated mortality to CVD risk during periods of extremely cold temperatures, potentially missing the delayed effects. Liu, Yavar and Sun noted that deaths in such studies may result from the challenge of adapting to abrupt temperature drops rather than a pure "cold effect." This implies that individuals without a history of CVD or comorbidities are less likely to exhibit fatal effects from short-term cold temperatures [76]. Additionally, prior research lacked an exploration of the relationship between cold temperature and CVD mortality based on continuous variables. By investigating the T_*MP*_ representing temperature pattern over a 30-year period (1988–2017) as a potential risk factor for CVDs, this was considered more representative of the accumulated and delayed effects of climate-patterned temperature (T_*MP*_) preceding any CVD incidence in 2017. The data analyses explored the statistical relationship between T_*MP*_ and CVD incidence, challenging the notion that low temperature is merely a short-term precipitating factor.

We have comprehensively incorporated the impacts of global warming into our data analyses concerning the T_*MP*_-CVD relationship. It's crucial to recognize that global warming does not uniformly elevate temperatures in every country and at all times. The extent of warming varies geographically and temporally, with some countries experiencing significantly different patterns of temperature change [77,78].

Given prior research indicating more pronounced lagged effects in low temperatures, it is probable that the underestimation is more significant for cold conditions than for heat [[79], [80], [81], [82]]. This potential discrepancy is reflected in our scatter plots, demonstrating a non-linear T_*MP*_- CVD correlation without distinct platform or any J-, V-, or U-shaped patterns (Fig. 1). This may suggest that there was no threshold T_*MP*_ worldwide, which is contrary to previous study findings [17,51,83,84]. Additionally, the shape of the trendline indicates a lack of bias in data quality. Furthermore, Earth's temperature has been rising due to global warming, coinciding with an overall decline in both CVD incidence and mortality worldwide [3,4]. This suggests that regional heatwaves may pose a significant risk for CVD mortality in limited areas for short periods. However, the risk from heatwaves may be minimal compared to the overall benefits of Earth's temperature increase for decreasing CVD mortality and incidence.

This study has provided a comprehensive analysis of a range of variables when exploring the correlation between T_*MP*_ and CVD incidence rate. Adjustments for humidity, but also other well-established major confounders, such as ageing [50,52], GDP PPP [16,52,53], obesity [[12], [13], [14],^52^] and urban living [10,11,55] were made. In addition to the independent predicting effects of T_*MP*_ on CVD incidence, simple and multiple regression were also applied to quantify the statistical explanation of T_*MP*_ on CVD incidence. For instance, simple regression (scatter plots), revealed that, worldwide, T_*MP*_ explained 46.94 % of CVD variance (R^2^ = 0.4694, Fig. 2.2). Multiple regression identified that T_*MP*_ was placed the second most important variable increasing R^2^ to 0.731 (Table 2, Table 3). This suggested that T_*MP*_ added 14 % of explanation for CVD incidence variation after ageing (59.1 %).

T_*MP*_ was strongly correlated to CVD in high-income countries compared to LMIC. This showed that T_*MP*_ may not be significant contributor to the WHO estimate that LMIC countries account for 75 % of CVD deaths worldwide [2]. The inverse correlation of T_*MP*_ to CVD incidence was also observed in different country groupings. There is a growing interest in examining the different contributing effects of low temperature on CVDs in LMIC and high-income countries [2]. In this study, both the bivariate correlations (Pearson's r and non-parametric) revealed that T_*MP*_ displayed a stronger correlation to CVD incidence in high-income countries compared to LMIC (z = 1.96 and 2.28, p < 0.050 respectively). This may be explained by emerging economies in LMIC over the past 30–40 years alleviating the detrimental effects of low T_*MP*_ on CVD pathogenesis. For example, heating systems and access to better healthcare service has become more affordable and available to greater numbers of populations in LMIC countries.

## 5 Strengths and limitations

Our study uniquely contributes to the literature regarding the correlation between cold spells and CVDs.

1. Instead of temporal temperature variations, “climate-patterned temperature” measured with long-term pattern of mean temperature value (30 years, T_*MP*_) has been correlated to CVD incidence [41,42]. This variable offers valuable insights into the long-term regional climate playing a crucial role in comprehending climate dynamics, evaluating the potential impacts of climate change, and making well-informed decisions in the field of public health such as CVDs.
2. Almost all previous studies into the temperature-CVD relationship included CVD mortality rate as the dependent variable. However, CVD mortality as an end point measure may not be an accurate indicator of CVD development risk. By using CVD incidence to explore the temperature-CVD relationship instead of CVD mortality, we potentially increase the accuracy of our findings in this study relating to CVD development risk.
3. The ecological study approach adopted in this study offered the advantage of incorporating more variables for data analysis compared to individual-based studies. Leveraging this, we included 5 potential confounding factors in our analyses to elucidate the independent role of T_*MP*_ in predicting CVD incidence. While the variables explained a large majority of CVD variance (74.7 %), residual variance remains unexplained, constrained by data availability and quality, limiting the inclusion of additional variables in our analyses.
4. Instead of referencing particular cities or other limited regions, this study quantified the worldwide role of climate-patterned temperature in determining CVD incidence risk depending on the potential confounding factors (humidity, ageing, GDP PPP, obesity and urbanization) and independent of these factors.

However, there are several important limitations to note.

1. It is neither ethical nor logistically feasible to randomize populations to different levels of climate-patterned temperature (T_*MP*_), socioeconomic status, ageing, humidity, obesity and urbanization to determine which population would have greater CVD incidence rate. The nature of the study approach based on the population level cross-sectional dataset makes this subject to two intrinsic fallacies: 1) Correlation does not imply causation; 2) The correlations observed in this study may not necessarily hold true at an individual level.

However, this study has attempted to obtain accurate correlation between T_*MP*_ and CVD incidence through accessing and incorporating more variables for analysing their intrinsic relationships. For instance, consideration of metrological science and incorporated long-term climate-patterned temperature (T_*MP*_), instead of short-term temperature changes as the independent variable. Additionally, several potential major confounding variables for analysing if and how much they affected the relationship between T_*MP*_ and CVD incidence were considered.

2. The data included in this study are the estimates of a broader population that cannot be entirely sampled. Therefore, the population level data published by IHME, WHO, United Nations and World Bank may be crude. Additioanlly, random errors may have been present in the collection and aggregation of the original data.
3. The genetic background of CVD is significant and may heavily influence CVD incidence. However, due to data unavailability, the confounding effects of genetic background while we analysed the T_*MP*_- CVD incidence relationship could not be excluded.
4. There are different sub-types of CVDs, and they may be affected by low temperature in different ways. We could not differentiate sub-types for analysis of their individual correlations to T_*MP*_*.*

## 6 Conclusion

This study suggested that low climate-patterned temperature (long-term pattern of low mean temperature, T_*MP*_) may be a significant and independent risk factor for CVD incidence worldwide. It also revealed that low T_*MP*_ may be a more significant contributor for increasing CVD incidence rate in high-income countries. Thus, climate-patterned temperature should be incorporated for studying the effects of temperature on CVD prediction for optimal results.

## CRediT authorship contribution statement

**Wenpeng You:** Conceptualization, Data curation, Formal analysis, Funding acquisition, Investigation, Methodology, Project administration, Resources, Software, Validation, Visualization, Writing – original draft, Writing – review & editing. **Jacob Sevastidis:** Conceptualization, Formal analysis, Funding acquisition, Investigation, Resources, Validation, Visualization, Writing – review & editing. **Frank Donnelly:** Conceptualization, Funding acquisition, Investigation, Resources, Validation, Visualization, Writing – review & editing.

## Ethics approval and consent to participate

All the variables incorporated into this study were freely extracted from the websites of international organizations, such as United Nation agencies and IHME. The whole set of the data involve the use of non-identifiable, pre-existing data about human beings. The individual person or their community cannot be traced as per the data. Therefore, they carry negligible ethical risk and there is no requirement for ethical approval or written informed consent from participation.

## Availability of data and materials

All the data included in our data analyses are freely available from the United Nation agencies' online repositories which are open to the public. The data sources have been described in the section of “Materials and Methods”. There is no need to obtain formal permission to use the data for non-commercial purpose which is compliant with the agency's public permission in their terms and conditions.

## Consent for publication

The authors approved the final manuscript for publishing.

## Funding source

This research did not receive any specific grant from funding agencies in the public, commercial, or not-for-profit sectors.

## Declaration of competing interest

The authors declare no conflict of interest.

## List of abbreviations

T_*MP*_: Climate-patterned temperature, calculated with long-term pattern of lower mean value of temperature for 30 years in this study
CVD: Cardiovascular disease
GDP PPP: Gross domestic product (purchasing power parity)
Life e [65]: Life expectancy at 65 years old
UN: United Nations
WHO: World Health Organization
IHME: Institute for Health Metrics and Evaluation of University of Washington
